# Antibody-drug conjugates in lung cancer: dawn of a new era?

**DOI:** 10.1038/s41698-022-00338-9

**Published:** 2023-01-11

**Authors:** Niamh Coleman, Timothy A. Yap, John V. Heymach, Funda Meric-Bernstam, Xiuning Le

**Affiliations:** 1 Department for Investigational Cancer Therapeutics (Phase I Program), University of Texas MD Anderson Cancer Center, 1515 Holcombe Blvd, Houston, TX 77030 USA; 2 Department of Thoracic/Head and Neck Medical Oncology, University of Texas MD Anderson Cancer Center, 1515 Holcombe Blvd, Houston, TX 77030 USA; 3 Institute for Applied Cancer Science, The University of Texas MD Anderson Cancer Center, Houston, TX USA; 4 Khalifa Institute for Personalized Cancer Therapy, MD Anderson Cancer Center, Houston, TX USA; 5 Department of Surgical Oncology, MD Anderson Cancer Center, Houston, TX USA

**Keywords:** Drug development, Targeted therapies

## Abstract

Antibody-drug conjugates (ADCs) are one of fastest growing classes of oncology drugs in modern drug development. By harnessing the powers of both cytotoxic chemotherapy and targeted therapy, ADCs are unique in offering the potential to deliver highly potent cytotoxic agents to cancer cells which express a pre-defined cell surface target. In lung cancer, the treatment paradigm has shifted dramatically in recent years, and now ADCs are now joining the list as potential options for lung cancer patients. Since 2020, the first ADC for NSCLC patients has been FDA-approved (trastuzumab deruxtecan) and two ADCs have been granted FDA Breakthrough Therapy Designation, currently under evaluation (patritumab deruxtecan, telisotuzumab vedotin). Furthermore, several early-phase trials are assessing various novel ADCs, either as monotherapy or in combinations with advanced lung cancer, and more selective and potent ADCs are expected to become therapeutic options in clinic soon. In this review, we discuss the structure and mechanism of action of ADCs, including insights from pre-clinical work; we summarize the ADCs’ recent progress in lung cancer, describe toxicity profiles of ADCs, and explore strategies designed to enhance ADC potency and overcome resistance. In addition, we discuss novel ADC strategies of interest in lung cancer, including non-cytotoxic payloads, such as immunomodulatory and anti-apoptotic agents.

## Introduction

Antibody-drug conjugates (ADCs) are, arguably, the fastest-growing class of oncology drugs in development, and while not a new concept, the potential to change clinical practice is vast. In lung cancer, the treatment paradigm has shifted dramatically in recent years, and now incorporates targeted therapy, immunotherapy, and systemic chemotherapy, and ADCs are now joining the list as potential options for lung cancer patients.

ADCs are unique in offering the potential to deliver highly potent cytotoxic agents to cancer cells that express a pre-defined cell surface target, thereby harnessing the powers of both cytotoxic chemotherapy and targeted therapy. Thus, ADCs are agents of precision oncology, and using these targeting properties one can greatly enhance the therapeutic index of the attached payload, compounds that would otherwise be too toxic for use. Comprising of three key components, ADCs are the “homing missiles” of modern drug development, and include (1) a monoclonal antibody that binds selectively to an antigen on the tumor cell surface, (2) a cytotoxic drug payload, and (3) a cleavable or non-cleavable linker^1,2^. To date, twelve ADCs have been granted FDA approval in oncology (Table 1), and with nine of these approved since 2017, the pace of development of this class is only accelerating.

**Table 1:** FDA-approved antibody-drug conjugates (ADCs) available in the clinic.

ADC	TargetmAb antigen isotype	Linker type	Payload	Payload class	Payload action	Disease indication	Year of Approval
Gemtuzumab ozogamicin (Mylotarg)	CD33IgG4	Cleavable	Ozogamicin	Calicheamicin	DNA cleavage	Relapsed or refractory CD33 + AML^**^	2000
Brentuximab Vedotin (Adcetris)	CD30IgG1	Cleavable	MMAE	Auristatin	Microtubule inhibitor	Relapsed or refractory systemic ALCL or classical HL Relapsed and/or refractory primary cutaneous ALCL or CD30 + MF (2017) classical HL, systemic ALCL or CD30 + PTCL^#^	2011 2018
Ado-trastuzumab emtansine (TDM1) (Kadcyla)	HER2IgG1	Non-cleavable	DM1	Maytansinoid	Microtubule inhibitor	Advanced-stage HER2 + breast cancer previously treated with trastuzumab and a taxane; early-stage HER2 + breast cancer in patients with residual disease following neoadjuvant trastuzumab–taxane-based treatment	2013 2019
Inotuzumab ozogamicin (Besponsa)	CD22IgG4	Cleavable	Ozogamicin	Calicheamicin	DNA cleavage	Relapsed or refractory B-ALL	2017
Fam-trastuzumab deruxtecan-nxki (T-DXd) (Enhertu)	HER2IgG1	Cleavable	DXd	Camptothecin	TOP1 inhibitor	Advanced-stage HER2 + breast cancer after two or more anti-HER2-based regimens: locally advanced or metastatic gastric cancer who have received a prior trastuzumab-based regimen; locally advanced or metastatic NSCLC patients who have progressed on platinum-based chemotherapy	2019 2021
Polatuzumab vedotin-piiq (Polivy)	CD79bIgG1	Cleavable	MMAE	Auristatin	Microtubule inhibitor	Relapsed or refractory DLBCL^¶^	2019
Sacituzumab govitecan-hziy (Trodelvy)	TROP2IgG1	Cleavable	SN-38 (active metabolite of irinotecan)	Camptothecin	TOP1 inhibitor	Metastatic triple-negative breast cancer in the third-line setting or beyond; metastatic urothelial cancer following progression on platinum-containing chemotherapy and a PD-1 or PD-L1 inhibitor	2020 2021
Enfortumab vedotin-ejfv (Padcev)	Nectin 4 IgG1	Cleavable	MMAE	Auristatin	Microtubule inhibitor	Advanced-stage urothelial carcinoma, following progression on a PD-1 or PD-L1 inhibitor and platinum-containing chemotherapy	2020
Belantamab mafodotin-blmf (Blenrep)	BCMAIgG1	Non-cleavable	MMAF	Auristatin	Microtubule inhibitor	Relapsed and/or refractory multiple myeloma in the fifth-line setting or beyond	2020
Tisotumab vedotin-tftv) (Tivdak)	Tissue IgG1 Factor	Cleavable	MMAE	Auristatin	Microtubule inhibitor	Recurrent or metastatic cervical cancer, no more than two prior systemic regimens in the recurrent or metastatic setting	2021
Loncastuximab tesirine-lpyl (Zynlonta)	CD20 IgG1	Cleavable	SG3199	PBD DIMER	DNA cleavage	Relapsed and/or refractory large B-cell lymphoma after 2 or more lines of systemic therapy, including DLBCL not otherwise specified, DLBCL arising from low grade lymphoma, and high-grade B-cell lymphoma	2021
Moxetumomab pasudotox (Lumoxiti)	CD22 IgG1	Cleavable	PE38	Pseudomonas exotoxin	Immunotoxin	Relapsed and/ or refractory hairy cell leukemia	2018

*AML* acute myeloid leukemia, *B-ALL* B-cell acute lymphoblastic leukemia, *BCMA* B-cell maturation antigen, *HL* Hodgkin lymphoma, *DAR* drug-to-antibody ratio, *DLBCL* diffuse large B-cell lymphoma, *mAb* monoclonal antibody, *MF* mycosis fungoides, *MMAE* monomethyl auristatin E, *MMAF* monomethyl auristatin F, *ALCL* anaplastic large cell lymphoma, *PTCL* peripheral T-cell lymphoma, *PBD* pyrrolobenzodiazepine, *TOP1* topoisomerase I, *TROP2* tumor-associated calcium signal transducer 2.

**Used as monotherapy or as a combination strategy with cytarabine and daunorubicin.

^#^Used (1) in combination with doxorubicin, vinblastine, and dacarbazine (AVD) for newly diagnosed classical HL, (2) as a combination strategy with cyclophosphamide, doxorubicin, and prednisone (CHP) for systemic anaplastic large cell lymphomas or (3) CD30+ Peripheral T-cell lymphoma.

^¶^Used as a combination strategy together with rituximab and bendamustine.

In this review, we discuss the structure and mechanism of action of ADCs, including insights from pre-clinical work; we explore the activity in lung cancer and summarize the recent progress of ADCs in the clinic (Table 2), describe current challenges and toxicity profiles of these compounds; finally, we explore potential combination strategies and other strategies designed to enhance ADC potency and overcome resistance.

**Table 2:** Summary of current key antibody-drug conjugate (ADC) clinical data in metastatic NSCLC.

Trastuzumab deruxtecan (NCT03505710) in HER2-mutant tumors	
Linker type - cleavable tetrapeptide-based linker Antibody subclass - IgG1	
FDA-approval - August 11 2022 On August 11, 2022, for NSCLC patients with activating human HER2 mutations, as detected by an FDA-approved test, and who have received a prior systemic therapy.	
Outcome	Trastuzumab deruxtecan (*n* = 91)
ORR (95% CI)	55% (44-65)
CR	1 (1%)
PR	49 (54%)
SD	34 (37%)
PD	3 (3%)
Median DOR, months (95% CI)	9.3 months (5.7–14.7)
Median PFS, months (95% CI)	8.2 (6.0–11.9)
Median OS, months (95% CI)	17.8 (13.8–22.1)
Median time to response, months (range)	1.5 (1.2–9.3)

Trastuzumab deruxtecan (NCT03505710) in tumors with HER2 overexpression		
Antibody subclass–IgG1 Linker type - cleavable tetrapeptide-based linker		
Outcome	Total population (*n* = 49)	
ORR (95% CI)	24.5% (13.3–38.9)	
Median PFS, months (95% CI)	5.4 (2.8–7.0)	
Median OS, months (95% CI)	11.3 (7.8-NE)	
	IHC 2 + (*n* = 39)	IHC 3 + (*n* = 10)
ORR (95% CI)	25.6% (13.0–42.1)	20% (2.5–55.6)
SD	41%	60%
DCR	66.7% (49.8%–80.9%)	80.0% (44.4%–97.5%)
DOR, months (range)	5.8 (3.2-NE)	6.0 (NE-NE)
Patritumab deruxtecan (NCT03260491)		
Outcome	Patritumab deruxtecan 5.6 mg/kg (*n* = 56)	
ORR (95% CI)	25% (14.4–38.4)	
CR	2%	
PR	23%	
SD	45%	
Median DOR, months (range)	7 (3–7)	

Datopotamab-deruxtecan (NCT03401385)			
Antibody subclass–IgG1 Linker type - cleavable tetrapeptide-based linker			
Outcome	4 mg/kg (*n* = 40)	6 mg/kg (*n* = 39)	8 mg/kg (*n* = 80)
ORR (95% CI)	23% (*n* = 9)	21% (*n* = 8)	25% (*n* = 20)
Confirmed CR/ PR	*n* = 7	*n* = 6	*n* = 19
PD	15%	21%	9%
DCR	73%	67%	80%

Telisotuzumab vedotin (NCT03539536)				
Antibody subclass–IgG1 Linker type - Cleavable dipeptide				
Outcome	EGFR mutant (*n* = 37)	Non-squamous EGFR WT cohort (*n* = 37)	c-Met–intermediate (*n* = 13)	c-Met–high (*n* = 13)
ORR (95% CI)	13.3% (3.8–30.7)	35.1% (20.2–52.5)	25% (9.8–46.7)	53.8% (25.1–80.8)

*ADC* antibody-drug conjugate, *CR* complete response, *DCR* disease control rate, *DOR* duration of response, *NE* not estimable, *NSCLC* non-small cell lung cancer, *ORR* objective response rate, *OS* overall survival, *PD* progressive disease, *PFS* progression-free survival, *PR* partial response, *SD* stable disease.

## Structure

The use of ADCs in animal models was first reported in the 1960s, but it was not until the 1980s that the first clinical trials with ADCs based on mouse immunoglobulin G (IgG) molecules were undertaken^3,4^. It has taken over fifty years of research for the initial promise to come to pass–the approvals of second-generation ADCs brentuximab vedotin (Seattle Genetics, developed in 2011) and trastuzumab emtansine (also known as ado-trastuzumab emtansine or TDM1, developed by Roche in 2013), have paved the way for the current plethora of clinical trials investigating potential ADCs in the clinic. Many of the recent next-generation ADCs have impressive activity against treatment-refractory cancers, and while limitations remain, such as toxicities related to treatment, inadequate biomarker selection, and acquired resistance, there is a reason for optimism for this therapeutic approach.

**Fig. 1:**
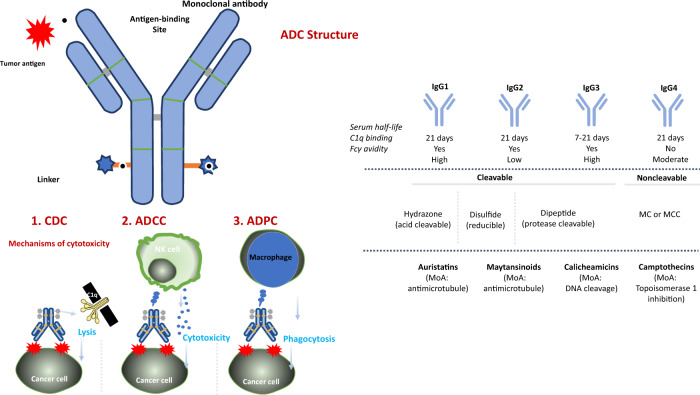
Structure and components of typical antibody-drug conjugates, including monoclonal antibodies, linkers, and payloads. Antibody: chimeric/humanized monoclonal IgG antibody targeting a protein preferentially expressed on the tumor cell surface. Linker: ensures payload is attached to antibody in plasma but is efficiently released in tumor cells. Linkers can be cleavable (via tumor-associated factors) or non-cleavable (lysosomal degradation); payload: enhances cytotoxicity, although variable drug:antibody ratio affects efficacy and clearance. The antibody component of ADCs engage with immune effector cells to elicit antitumor immunity, including complement-dependent cytotoxicity (CDC), antibody-dependent cell cytotoxicity (ADCC), and antibody-dependent cell phagocytosis (ADCP) effects. MC maleimidocaproyl, MCC maleimidomethyl cyclohexane-1-carboxylate; MoA mechanism of action.

As a class of drugs, all ADCs are composed of three principal components: an antibody that binds a tumor-associated antigen, a cytotoxic payload, and a linker that connects the two (Fig. 1). Each of these three components can differ between different ADCs, which may lead to contrasting pharmacological and clinical properties.

## Mechanism of action

**Fig. 2:**
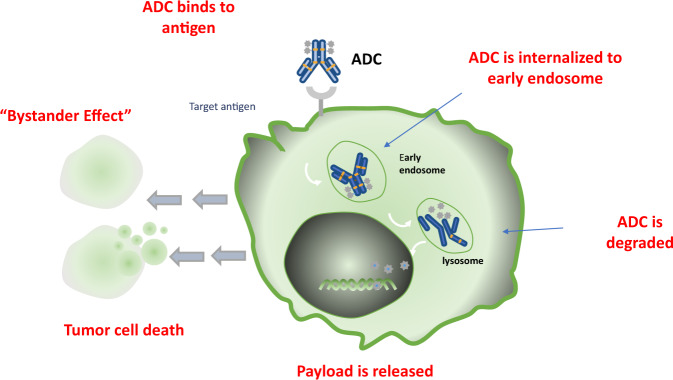
Mechanisms of action of antibody-drug conjugates. ADCs selectively deliver toxic payload to tumor cells resulting in cell death. The “bystander effect” of ADCs alter stumor microenvironment to enhance cell killing.

The mechanism of action of ADCs is complex, involving the binding of antibodies to the target antigen, subsequent internalization, linker breakdown, and intracellular payload release, and while this appears a simple process, the reality is more complicated. In contrast to other drugs in modern cancer therapy, ADCs require the action of cancer cells for prime effectiveness. ADCs are agents of precision medicine - “biological homing missiles” which can specifically target tumor cells and induce cell death, allowing for increased drug delivery to specifically selected tumor cells, with the benefit of reduced off-target events. In ADCs, the monoclonal antibody binds to the target antigen specifically expressed on the tumor cell; the ADC is then internalized by tumor cells to ultimately fuse with lysosomes, allowing for the release of the cytotoxic which ultimately leads to cell death or apoptosis of cells by targeting DNA or microtubules (Fig. 2).

Some payloads exert a bystander effect, where the free drug is released unintentionally from the target tumor cell across the cell membrane following internalization and degradation of the ADC, to kill adjacent tumor cells, including those cells that may not express the target antigen on its cell surface. This feature of certain ADCs is often debated and some pharmacological characteristics, such as a hydrophobic payload or a cleavable linker, appear to play a major role in this phenomen^5^. When the cytotoxic payload released is permeable or transmembrane, it can also induce the ‘bystander effect’, which can enhance the efficacy of ADC. In addition, the bystander effect of ADCs may also alter the tumor microenvironment, which in turn may further enhance the killing effect of ADCs^6^. Moreover, regarding the bystander effect, some chemical properties of the payloads, e.g., lipophilic, hydrophobic, and uncharged payloads are important for the membrane permeability of ADCs, allowing for the distribution from the ADC-targeted tumor cells to non-targeted tumor cells^5^. As described above, the linker type, such as the conjugation to the lysine residue, can also result in attenuated bystander effect, as charged residues can promote drug staying within the targeted tumor cell^5^. Important considerations in the design of an ADC include target cell selection, the nature of antigen, structure, and stability of the antibody, the linker chemistry, and the cytotoxic payload.

### Antibody and antigen

The choice of the antigen and the selection of the appropriate antibody is an essential part of ADC design. Firstly, the antibody is selected depending on the molecular target. It is preferable that the antibody recognizes an overexpressed target only at the tumor site to avoid delivering the payload inappropriately to non-target sites, as ADCs are designed to deliver their toxic payload to any cell which expresses the target antigen. (Erb-B2 Receptor Tyrosine Kinase 2) (*ERBB2* or *HER2*), for example, is expressed more than 100 times in tumor tissues in comparison to non-cancer tissue^7,8^. TROP2 and nectin 4 are further clinical examples^9,10^, and each of these proteins, while being expressed to some degree in non-cancerous tissues, are overexpressed by tumor cells by a significantly increased number^9,11,12^. Secondly, the antigen at which the antibody is directed at should be present in high copy number on the cancer cell (>105/cell)^13^. Human IgGs encompass four subclasses—IgG1, IgG2, IgG3, and IgG4—and these differ in their constant domains and hinge regions; in addition, there are subtle variations between subclasses which affect the solubility and half-life of antibodies, as well as their affinity for different Fcγ receptors expressed on immune effector cells^14,15^. The majority of modern ADCs are developed based upon the IgG1 architecture, since compared to the other subclasses, IgG1 optimally combines solubility, a long serum half-life, and binding affinity Fcγ receptors^16^.

Other biological processes also affect ADC activity including rates of target turnover, internalization, lysosomal processing, and degradation. For example, higher rates of target turnover can cause more efficient drug delivery and target replenishment which can lead to increased anti-tumor activity^17^. Moreover, oncogenic targets, targets only present on the surface of cancer cells, are less likely to have downregulation of expression as a mechanism of drug resistance and so can be exploited for additional ADC activity^18^. Tumor heterogeneity is another consideration: in HER2-positive breast cancer, in contrast to patients with homogeneous HER2 expression, patients with high levels of intratumor or inter-tumor heterogeneity have inferior responses to ado-trastuzumab emtansine (TDM1, trastuzumab-DM1)^19^.

### Linker

Linkers connect the antibody to the payload and are a key factor related to the stability of ADC and payload release profiles. Linkers are classified as cleavable and non-cleavable based on their cleavage mechanisms^20,21^, and are important for the eventual therapeutic index of the ADC^20^. Cleavable linkers are designed to break down and release the cytotoxic payload of the ADC in response to factors associated with the tumor; thus, by using the disparities between tumor cells and the systemic circulation, the payload is released in a guided, precise way. Cleavable linkers are divided into two classes, chemical (which include disulfide and hydrazone bond) and enzyme linkers (peptide and glucuronide bond)^20^. Hydrazone is a pH-sensitive linker, which allows for the ADC to remain stable in the systemic circulation and is hydrolyzed at specific pH (e.g., pH 4.8 induces release of the payload in lysosomes^20,22^. The degradation of cleavable linkers, therefore, can vary depending on a number of specific features, either outside or inside the target cell, such as external pH (acid-labile linkers), specific lysosomal proteases (protease-cleavable linkers) or glutathione reduction (disulfide linkers)^23^. Recent cleavable linkers (e.g., enfortumab vedotin, sacituzumab govitecan, trastuzumab deruxtecan (T-DXd))^21^ are stable in the systemic circulation, in contrast to non-cleavable linkers, e.g., ado-trastuzumab emtansine (TDM1).

Non-cleavable linkers, meanwhile, are comprised of stable bonds which are more resistant to proteolytic cleavage in blood; following the internalization of ADCs by lysosomes or proteases, and subsequent antibody degradation, cleavage then occurs which then leads to release of the payload^23^. TDM1, for example, is an ADC that utilizes a non-cleavable linker (thioether-based), and is comprised of an anti-HER2 monoclonal antibody linked with DM1 (mertansine) via the thioether-based linker^24^, The non-cleavable linker in TDM1 allows the ADC to remain stable in the systemic circulation and then releases the active drug, the metabolite of DM1, lysine-MCC-DM1, following antibody degradation^20^. In non-cleavable linkers, such as the conjugation to the lysine residue, some charged residues can promote drug staying within the targeted tumor cells, resulting in attenuating the bystander effect.

### Payload

The payload is the portion of the ADC that exerts potent cytotoxicity on the tumor cell when internalized. To be used as payloads, high potency, with IC50 in nanomolar and picomolar range, is required; in addition, they should be stable in physiological conditions and have available function groups for conjugation with the antibody^20,25^. Modern ADC payloads can be broadly divided into four main classes – auristatins (anti-microtubules), maytansinoids (anti-microtubules), calichaemicins (DNA cleavage), and camptothecins (topoisomerase 1 inhibition) (Fig. 1). Generally, these payloads act on either the DNA structure, and induce cell death by apoptotic mechanisms (topoisomerase 1 inhibitors, calichaemicins)^26^, or affect the microtubule structure of the cell (auristatins and maytansinoids), inducing G2/M arrest and apoptosis by inhibition of microtubule polymerization. Regardless of class, these agents are generally potent cytotoxic compounds which tend to be characterized by an IC_50_ in the nanomolar and picomolar range, which if given systemically would cause severe toxicities^2^. The cytotoxic payloads of almost two-thirds of ADCs that are currently being tested in clinical trials are based on either auristatins or maytansinoids^27^. Monomethyl auristatin E (MMAE)^28^, for example, is a microtubule destabilizer which has been incorporated into several ADCs such as brentuximab vedotin and telisotuzumab vedotin. Maytansinoids, such as DM1, bind to tubulin and disrupt microtubule instability^29^.

Non-cytotoxic payloads are now emerging, and one such example under investigation are immunomodulatory payloads. Termed immune-stimulating antibody conjugates (ISACs), these agents aim to promote tumor regression and induce tumor anti-immunity^30^. ISACs include non-cytotoxic payloads which activate myeloid antigen-presenting cells, stimulating the immune system with a different mechanism than checkpoint blockade. SBT6050 is one such example, which targets HER2 with a toll-like receptor (TLR) 8 agonist payload^31^. TLRs are a skillful system of receptors in the innate immunity that play an important role in the interplay between tumor and innate immunity^32^. Activation of TLR7 and/or TLR8 can cause a cascade of signal pathways to become induced, which can in turn activate, for example, NF-κB which leads to the secretion of cytokines and chemokines, and causes activation of lymphocytes^20,32^. BDC-1001 is a novel HER2-targeting TLR7/8 ISAC that is currently under investigation in the setting of early phase clinical trials (NCT04278144)^33^, both as monotherapy and in combination with immune checkpoint inhibitors in patients with advanced HER2-driven cancers. Indeed there are many current ongoing clinical trials using immune checkpoint inhibitors in combination with ADCs, in an effort to increase efficacy of these agents and potentially boosting immunotherapy activity^34^. For example, the ImmunoTAC platform has been developed by Silverback Therapeutics; this includes a number of ISACs which use TLR8 agonists as payloads (e.g., SBT6050, SBT6290, and SBT8230)^35^. Stimulator of interferon genes (STING) agonists are another novel payload^36^, and XMT-2056 from Mersana and CRD5500 from Takeda are examples of prominent STING-agonist ADC programs being developed clinically^37^.

#### Drug-to-antibody ratio

The drug-to-antibody ratio (DAR) is the average number of payload moieties attached to each monoclonal antibody. This property varies between ADCs and informs the pharmacology and activity of the ADC^38^. Ongoing efforts are now focused on activities that may enhance the activity of the ADC, by exploiting the structure of the molecule; increasing the DAR is one such example, increasing the bystander effect is another strategy being explored. Camptothecins, such as sacituzumab govitecan, are an example where increasing the DAR has been exploited as a successful strategy. Sacituzumab govitecan is an ADC which conjugates an anti-Trop2 antibody with SN-38, the active metabolite of irinotecan, which is almost 1000 times more active than irinotecan, and consequently, due to its toxicity and poor solubility, cannot be delivered as an unbound drug. Fam-trastuzumab deruxtecan-nxki is another example of a promising highly active ADC in the clinic, with a high DAR of 8 which is likely driving its activity^39^.

## Established ADC targets in lung cancer

### HER2

Human epidermal growth factor receptor 2 (HER2) is a transmembrane protein encoded by the erb-b2 receptor tyrosine kinase 2 (ERBB2) gene, which belongs to the ErbB or epidermal growth factor receptor (EGFR) family^40^. Already an established target in breast and gastric cancer, HER2 overexpression is also associated with a large variety of cancers, such as lung, ovarian, colorectal, and salivary gland tumors^41^. HER receptors exist as both monomers and dimers, either homo- or heterodimers, and ligand binding to HER1, HER3, or HER4 induces rapid receptor dimerization^42^. While HER2 has no identified ligand, it is the preferred partner to form heterodimer with other HER members, which results in activation of the HER signaling pathways^43^. In NSCLC, documented HER2 alterations include HER2 gene amplification, HER2 mutations, and HER2 protein overexpression. The frequency of HER2 protein overexpression varies considerably in the literature, though it has reportedly been observed in up to 20% of patients with NSCLC^44,45^, and correlates with inferior survival outcomes^46,47^. Targetable HER2 mutations occur in ~2% of advanced NSCLC cases.

#### Trastuzumab emtansine

TDM1 is an ADC comprising an anti-HER2 monoclonal antibody trastuzumab linked via a non-cleavable thioether linker (maleimidomethyl cyclohexane-1-carboxylate (MCC)) to cytotoxic payload DM1 (emtansine). TDM1 has been approved since 2013 as a single agent for the treatment of metastatic HER2-positive breast cancer^48^, and has subsequently been investigated across tumors. In advanced NSCLC, using TDM1 in a phase II basket trial led to overall response rate (ORR) of 44% and median progression-free survival (PFS) of 5 months in patients with activating HER2 mutations; 39% of patients achieved stable disease. In addition, treatment was well tolerated, AEs were predominantly grade 1 or 2^49^. In spite of this, TDM1 is not yet FDA-approved in this setting, though it is included in the NCCN guidelines as a recommendation in advanced pre-treated NSCLC with activating HER mutations (evidence category 2 A).

#### Fam-trastuzumab deruxtecan (Enhertu, T-DXd)

Fam-trastuzumab deruxtecan (T-DXd) is an ADC that contains humanized anti-HER2 monoclonal antibody trastuzumab connected to the topoisomerase inhibitor deruxtecan (DXd) using a protease-cleavable peptide linker (DAR of 8)^39^. The combination of the cleavable linker, the membrane-permeable cytotoxic payload with high DAR, leads to increased activity on neighboring cells are all key features of the ADC structure, and allows for the use of T-DXd in cancers with heterogeneous HER2 expression, thus, lung cancer is a prime candidate for this compound. This observation was noted in pre-clinical models, where T-DXd proved efficacious in PDX models insensitive to TDM1^39^.

The phase I dose-escalation and dose-expansion study of T-DXd in patients with advanced HER2-expressing or HER2- mutant solid tumors included eighteen patients with NSCLC and included tumors with activating HER2 mutations or HER2 expression (defined by IHC ≥ 1+ or amplification by in-situ hybridization or next-generation sequencing)^50^. Ten of these patients achieved a partial response (ORR 55.6%), median progression-free survival (mPFS) was 11.3 months, and median duration of response (DOR) 10.7 months^50^. ORR was 72.7% (*n* = 8) in patients with HER2-mutant NSCLC, of whom six had insertions in exon 20; mPFS was 11.3 months and mDoR 9.9 months. Following these impressive data, advanced NSCLC patients with HER2 overexpression or HER2 activating mutations were enrolled on the single-arm international phase II DESTINY-Lung01 trial.

Interim analysis of the HER2-overexpressing cohort (overexpressing HER2 centrally confirmed, IHC 2+ or 3+) has shown preliminary evidence of antitumor activity in 49 heavily pre-treated patients with HER2-overexpressing NSCLC. Patients with stable brain metastases were included, 34.7% had CNS metastases at enrollment; 79.6% of patients had HER2 IHC 2+ and 20.4% had HER2 IHC 3+. Confirmed overall response rate (ORR) by ICR was 24.5% (95% CI, 13.3%–38.9%), including one complete response (CR). Response rates varied based on the expression of HER2: ORR for patients with IHC overexpression of 3+ was 20.0% (95% CI, 2.5%–55.6%) and 25.6% for IHC 2+ (95% CI, 13.0%–42.1%); IHC 3+, median DOR was 6.0 months (95% CI, 3.2-NE months). Disease control rate (DCR) was 69.4% (95% CI, 54.6%–81.8%); estimated median PFS was 5.4 months (95% CI, 2.8–7.0 months). Even more impressive responses were produced in the HER2-mutant NSCLC cohort of the DESTINY-Lung01 (NCT03505710) trial^51^. Confirmed objective response occurred in 55% of the patients (95% CI, 44–65), with one CR, stable disease (SD) ≥ 6 weeks in 34 patients (37%), DCR 92%. Responses observed were diverse, demonstrated in patients with both different HER2 mutation subtypes and across exon locations; in addition, responses were observed in patients with no detectable HER2 expression and in patients with no HER2 amplification. Median PFS was 8.2 months (95% CI = 6.0–11.9 months) and median OS was 17.8 months (95% CI = 13.8–22.1 months)^51^.

The safety profile was generally manageable, included ILD, though two cases of treatment-related death did occur, both ILD-related. Toxicities greater than grade 3 occurred in 46% of patients; the most documented of these included neutropenia, anemia, nausea, and fatigue (19%, 10%, 9%, and 7%, respectively). Observed toxicities were generally consistent with previously reported clinical trials and drug-related adverse events resulted in discontinuation of study drug in 25% of patients, including pneumonitis in 13% and ILD in 5% and drug-related ILD occurred in 26% of patients^51^.

On August 11, 2022, the Food and Drug Administration granted accelerated approval to T-DXd for NSCLC patients with activating HER2 (ERBB2) mutations, as detected by an FDA-approved test, and who have received a prior systemic therapy, representing the first drug approved for HER2-mutant NSCLC.

### HER3

HER3 is a member of the ErbB/HER protein kinase family, and while HER3 itself is not an oncoprotein and lacks tyrosine kinase activity, HER3 heterodimerizes with other RTKs to activate oncogenic signaling via the PI3K/AKT/mTOR pathway and also MEK/MAPK, Jak/Stat, Src kinase signaling leading to cell proliferation and ultimately the promotion of cancer cell survival, proliferation, and progression^52,53^. HER3 expression can also mediate resistance to targeted therapy (e.g., resistance to EGFR-targeted therapies in lung cancer, via maintenance of HER3-mediated activation of PI3K/AKT signaling^53,54^. Receptor tyrosine-protein kinase erbB-3 (HER3) is expressed across a variety of solid tumors and has been reported in 83% of primary NSCLC tumors^55,56^. HER3 expression is also associated with metastatic disease progression and decreased relapse-free survival in patients^57^, thus, HER3 is an attractive therapeutic target in NSCLC, especially due to its potential functional role in mediating resistance to targeted therapies.

#### Patritumab deruxtecan

Patritumab deruxtecan (U3-1402, HER3-DXd) is a novel HER3-directed ADC composed of a human immunoglobulin G1 monoclonal antibody to HER3 (patritumab) which is linked covalently to a topoisomerase I inhibitor payload (MAAA-1181a, an exatecan derivative) via a stable tetrapeptide-based cleavable linker with a high DAR ratio at 4^58,59^. The payload is highly potent with a short systemic half-life, and the cell membrane is permeable, which allows for a bystander killing effect, affecting both target and surrounding tumor cells^60^. Based on promising pre-clinical data, which demonstrated antitumor activity of HER3-DXd in multiple solid tumor xenograft models^61^, a phase I study was initiated in patients with locally advanced or metastatic EGFR-driven NSCLC with prior treatment with EGFR TKI and platinum-based chemotherapy (NCT03260491), which ultimately led to FDA breakthrough therapy designation (BTD) in December 2021.

In the reported dose-escalation part of the study, among 57 patients receiving HER3-DXd 5.6 mg/kg IV Q3W, the confirmed ORR was 39% (95% CI, 26.0–52.4), and median PFS was 8.2 (4.4–8.3) months, mDOR of 6.9 months^62^. Responses were observed in patients with known and unknown EGFR TKI resistance mechanisms and, responses were observed across a range of HER3 expression, such that HER3 expression levels did not clearly distinguish responders vs non-responders^62^. Patients who received prior osimertinib and platinum-based chemotherapy demonstrated similar efficacy to the overall population, as did patients with brain metastases^62^. The most common grade ≥3 TEAE were hematologic toxicities, which included thrombocytopenia (28%) and neutropenia (19%). Only four patients (5%) experienced ILD-related treatment, none of which were grade 4 or 5^62^.

Recently reported dose-expansion safety and activity data confirm that HER3-DXd at 5.6 mg/kg provides promising evidence of preliminary antitumor activity and safety in heavily pre-treated patients with advanced EGFR-driven NSCLC^63^. Most patients (*n* = 49, 86%) had received prior osimertinib, and patients with stable CNS metastases were included (*n* = 27, 47%). An ORR of 25% (14/56; 14.4–38.4) was observed, including one CR (1/56, 2%) PR in 13/56 (23%) and SD in 25/56 (45%)^63^. Interestingly, efficacy was observed across various mechanisms of EGFR TKI resistance, including EGFR C797S mutation, *MET* amplification, *HER2* mutation, *BRAF* fusion, and *PIK3CA* mutation. DCR of 70% was achieved (39/56, range 55.9–81.2) and DoR was 7 months (3.0–7.0).

Overall, the safety profile of HER3-DXd was manageable, and the most common grade ≥3 treatment-emergent adverse events were hematological. The frequency of treatment-related ILD (5%) was similar to the reported incidence in prior trials of EGFR-TKIs for patients with NSCLC (ILD rate, 0-5.7%)^64^. There were no deaths. The ongoing phase II HERTHENA-Lung01 study is currently evaluating HER3-DXd in patients with metastatic NSCLC following at least one EGFR TKI and one platinum-based chemotherapy.

### Trop2

Trophoblast cell surface antigen (Trop2) is a transmembrane glycoprotein calcium signal transducer that mediates cell migration and anchorage-independent growth, and is expressed across many epithelial tumors^65^. It has been associated with poor overall and disease-free survival in several types of solid tumors^66,67^. In lung cancer, Trop2 overexpression has been observed in up to 64% of adenocarcinoma and up to 75% of squamous cell carcinoma NSCLC^66,68^, and associated with reduced survival^69^; a potential role in resistance to chemotherapy and CD8+ T-cell apoptosis has also been suggested^70^.

#### Datopotamab-deruxtecan (Dato-DXd)

Dato-DXd is an ADC composed of a TROP2-directed monoclonal antibody conjugated to a potent topoisomerase I inhibitor via a stable tetrapeptide-based cleavable linker (DAR of 4). Dato-DXd showed encouraging antitumor activity in phase 1 TROPION-PanTumor01 trial (NCT03401385), an ongoing multicenter, open-label, dose-expansion study evaluating Dato-DXd in different dose levels in solid tumors^71^. The dose-escalation part of the trial assessed the safety and tolerability of increasing doses of Dato-DXd, while the dose-expansion of the trial is assessing the safety and tolerability of Dato-DXd using a selection of dose levels (4 mg/kg, 6 mg/kg, 8 mg/kg) in patients with NSCLC, and in patients with metastatic triple-negative breast cancer. Patients were not selected based on TROP2 expression.

Updated interim results for 159 NSCLC patients treated with different doses of Dato-DXd (4 mg/kg, 6 mg/kg or 8 mg/kg) were reported at the World Lung Cancer Conference 2021^72^; most had previously received immunotherapy and platinum-based chemotherapy, 84%, and 94%. ORR ranged from 21 to 25%, (23% (*n* = 9) at 4 mg/kg, 21% (*n* = 8) at 6 mg/kg, and 25% (*n* = 20) at 8 mg/kg). There was one confirmed CR (at 6.0 mg/kg), 32 PRs by BICR (8 PRs in 29 patients at 4.0 mg/kg, 4 PRs in 20 patients at 6.0 mg/kg, and 20 PRs in 76 patients at 8.0 mg/kg); 29 CRs or PRs were confirmed. Efficacy results supported durable clinical responses, DCR 67 to 80% was observed and a median PFS f4.3 to 8.2 months across doses, though longer follow-up is required^72^. 4/kg and 6 mg/kg doses were better tolerated and the most frequent grade 3 adverse events included anemia, stomatitis, mucosal inflammation, and fatigue. Unsurprisingly, patients treated at the higher dose of 8 mg/kg dose experienced higher frequency of adverse events: 14 cases (8%) of ILD were reported, and the majority of ILD cases (12/14) occurred in the 8 mg/kg cohort; 3 of these were fatal Grade 5 events^72^. The 6 mg/kg dose was identified as the recommended dose level for the registrational TROPION-Lung01 Phase III trial.

Encouraging results for NSCLC patients with actionable genomic alterations included on TROPION-PanTumor01 were reported at the 2021 ESMO Congress, which included 34 patients (median age, 62 years; 56% women) with advanced/metastatic NSCLC^73^; actionable genomic alterations reported by investigators included *ALK* (*n* = 3), *EGFR* (*n* = 29) and *ROS 1* and *RET* (both *n* = 1). Confirmed ORR across doses was 35% (95% CI, 19.7–53.5), median DOR was 9.5 months (95% CI, 3.3–NE).

#### Sacituzumab govitecan (SG)

Sacituzumab govitecan (SG, IMMU-132, Trodelvy®) is a first-in-class anti-Trop2 ADC, which consists of humanized anti-Trop2 monoclonal antibody sacituzumab linked to the topoisomerase I inhibitor SN-38 by a hydrolysable cleavable linker with high DAR ratio (7.6). In April 2020, SG was granted accelerated FDA-approval as a treatment for TNBC following at least two prior therapies for metastatic disease based on results from the phase I/II IMMU- 132-01 basket trial in treatment-refractory metastatic epithelial cancers^74^, and recently accelerated FDA-approval in urothelial carcinoma.

In the phase I/II IMMU-132-01 basket trial, patients were enrolled regardless of Trop2 expression and treated at doses ranging from 8 to 18 mg/kg on days 1 and day 8 in a 3-week cycle. NSCLC and SCLC were included: in the NSCLC cohort, 54 patients received 8, 10, or 12 mg/kg; ORR was 16.7% (7.9–29.3), there were 9 PRs (16.7%), 22 SDs (40.7%). mDOR 6.0 months (2.5–21.0), mPFS was 4.4 months, and mOS was 7.3 months^74^. 10 mg/kg dose was selected for further development in dose-expansion studies. In the NSCLC cohort, 59.6% experienced grade 3 or greater TRAEs, including neutropenia (42.4%), anemia (10.3%), diarrhea (7.9%), fatigue (6.3%), and febrile neutropenia (5.2%). In the SCLC cohort, a safe and effective therapeutic profile was confirmed in heavily pre-treated mSCLC patients, including those who are chemo-sensitive or chemo-resistant to first-line chemotherapy^75^. ORR in this cohort was 17.7%, mPFS of 3.7 months, mDOR of 5.7 months, and mOS of 7.1 months. Grade 3 higher TEAEs were comparable to other tumor types, and included neutropenia (34%), fatigue (13%), diarrhea (9%), hypoxia (4%), and febrile neutropenia (2%)^75^.

### MET

#### Telisotuzumab vedotin

Dysregulation of *MET* signaling via receptor overexpression has been implicated in the development of NSCLC^76,77^, as well breast, ovarian, colorectal, and prostate cancer^78–81^. Telisotuzumab vedotin (Teliso-V) is composed of anti–c-Met humanized mAb ABT-700 attached to MMAE via a valine-citrulline linker, and a phase I study confirmed the compound was well tolerated and demonstrated antitumor activity in c-Met+ NSCLC^82^.

In January 2022, FDA BTD was awarded to Telisotuzumab vedotin (Teliso-V) based on data from the LUMINOSITY trial (NCT03539536), an ongoing Phase II study in NSCLC patients with varying levels of c-Met expression in the second- or third-line setting. In patients with EGFR WT non-squamous NSCLC, ORR was 53.8% in the c-Met high group and 25.0% in the c-Met intermediate group. Teliso-V is now under evaluation in clinical trials in combination with osimertinib (phase I) (NCT02099058) in previously treated c-MET overexpressing NSCLC; it is also being investigated as monotherapy in patients with previously treated c-Met overexpressing NSCLC in the randomized Phase III study TeliMET NSCLC-01 (NCT04928846).

### ADC targets in development of lung cancer

There are several early-phase clinical trials actively assessing oncogenic targets-of-interest using novel ADCs in advanced lung cancer. Of note, there are currently no registered trials of ADCs in early-stage NSCLC, though this may only be temporary. Ongoing interest in advanced NSCLC include NECTIN4, Tissue Factor (TF), CEACAM5, mesothelin, and LIV1. These targets with associated ADCs in clinical trials are summarized in Table 3. Enfortumab-vedotin is an ADC targeting Nectin 4 (PVRL4), a member of a type 1 transmembrane protein family related to immunoglobulin-like adhesion molecules, which has demonstrated clinical benefit in advanced previously treated urothelial cancer^83^. This has led to the Food and Drug Administration (FDA) approval of enfortumab vedotin-ejfv in this setting, and this is currently under investigation in NSCLC (Table 3). Tisotumab vedotin-tftv, a tissue factor (TF)-directed antibody and microtubule inhibitor conjugate, is another ADC recently granted accelerated FDA-approval: this was based on the phase II innovaTV 204 trial^84^, in patients with previously treated recurrent or metastatic cervical cancer. Given that tissue factor expression has been shown to be higher in advanced NSCLC^85^, TF is another compelling target of interest, which is currently under investigation in NSCLC (Table 3).

**Table 3:** Ongoing antibody-drug conjugates for NSCLC in early-phase clinical trials.

Drug	Target	Payload	ClinicalTrials.gov (Study Name)	Other solid tumors treated in early-phase clinical trials
Trastuzumab Emtansine (TDM1)	HER2	Emtansine (DM1)	NCT02289833	Breast
Trastuzumab Deruxtecan (DS-8201)	HER2	Deruxtecan (DXd)	NCT04644227 (DESTINY-LUNG02)	Breast, gastric, gastro-esophageal, osteosarcoma, biliary tract, cervical, endometrial, ovarian, pancreas
ARX788HE	HER2	Monomethyl Auristatin F (MMAF)	NCT03255070 (ACE-Pan Tumor 01)	Breast, gastric
Patritumab Deruxtecan	HER3	Deruxtecan (DXd)	NCT04619004 (HERTHENA- Lung01)	Breast, colon, head & neck cancer
Enapotamab-Vedotin (HuMax-AXL-ADC)	AXL	Vedotin (MMAE)	NCT02988817	Ovarian, cervical, endometrial, thyroid, melanoma, sarcoma
CAB-AXL-ADC (BA3011)	AXL	Vedotin (MMAE)	NCT04681131	Pancreas, melanoma, sarcoma
CX2029	CD71	Vedotin (MMAE)	NCT03543813 (PROCLAIM-CX-2029)	Head & neck, diffuse large b-cell lymphoma, esophageal
Tusamitanib-Ravtansine (SAR408701)	CEACAM5	Ravtansine (DM4)	NCT04154956 (CARMEN-LC03) NCT04524689 (CARMEN-LC05)	Breast, pancreas
Mirvetuximab-Soravtansine (MIRV)	FRα	Soravtansine (DM4)	NCT01609556	Ovarian, endometrial, fallopian tube, primary peritoneal, breast
ELU-001 (FA-CDC)	FRα	C’Dot-Drug-Conjugate (CDC)	NCT05001282	Ovarian, endometrial, peritoneal, colorectal, gastric, esophageal, breast, cholangiocarcinoma, biliary duct
Datopotamab-Deruxtecan (DS-1062)	HER2	Deruxtecan (DXd)	NCT04656652 (TROPION- Lung01) NCT04484142 (TROPION-Lung05) NCT03401385 (TROPION-PanTumor01)	Breast (triple-negative, hormone receptor positive/HER2-negative breast cancer), urothelial, gastric, esophageal
RG7841 (DLYE5953A)	Ly6E	Vedotin (MMAE)	NCT02092792	Breast, pancreas, ovarian
Anetumab-ravtansine (BAY94-9343)	Mesothelin	Ravtansine (DM4)	NCT03102320	Mesothelioma, ovarian, pancreas, breast
Telisotuzumab vedotin (ABBV-399)	c-MET	Vedotin (MMAE)	NCT03539536	Solid tumors
Lifastuzumab-vedotin (RG- 7599, DNIB0600A)	NaPi2b	Vedotin (MMAE)	NCT01363947 NCT01995188	Ovarian
Upifitamab-Rilsodotin (XMT-1536)	NaPi2b	Rilsodotin (AF- HPA)	NCT03319628 (UPLIFT)	Ovarian
Enfortumab-vedotin (ASG- 22CE)	Nectin-4	Vedotin (MMAE)	NCT04225117	Urothelial, breast, head & neck, gastric, gastro-esophageal, esophageal, prostate, ovarian
Tisotumab vedotin (HuMax- TF-ADC)	Tissue Factor (TF)	Vedotin (MMAE)	NCT03245736 NCT01631552	Cervical, ovarian, endometrial, bladder, prostate, esophageal
Sacituzumab govitecan (IMMU-132, hRS7-SN-38)	Trop2	Govitecan (SN-38)	NCT03964727 (TROPiC S-03) NCT03337698 (Morpheus Lung)	Breast, head & neck, endometrial, gastric, esophageal, hepatocellular, ovarian, prostate, bladder, renal cell, cervical, pancreas, GBM
Naptumomab-estafentanox (NAP, ABR-217620, Anyara)	Staphlococcal enterotoxin A and 5 T4 (TPBG)	Immune-conjugate	NCT04880863 (NT-NAP-102–1)	Renal cell, pancreas
Cofetuzumab-pelidotin (ABBV-647, PF- 06647020)	PTK7	Pelidotin (Aur0101)	NCT04189614	Breast, ovarian
Trastuzumab-duocarmazine (SYD985)	HER2	Duocarmazine	NCT04235101	Breast, ovarian, endometrial
MORAb-202 (Farletuzumab Linked to Eribulin Mesylate)	Folate Receptor-α	Eribulin	NCT03386942	Solid tumors

## Toxicity

ADCs were developed with the intention of limiting toxicities by their design, however, managing toxicities related to ADCs in the clinic remains an ongoing challenge. While the safety profiles of modern novel ADCs are more favorable, ADCs can still cause several disabling, and potentially deadly toxicities. ADC dose-limiting toxicities can be wide-ranging, and have been shown to include hepatic, neurological and ophthalmic adverse events, which predominantly arise from off-target effects caused by the premature release of the ADC payload in circulation^86^, as well as from the ADC binding to non-cancerous expressor cells of the target antigen^86^. Trastuzumab deruxtecan, for example, has led to grade 5 ILDs across tumor types^87^, and the auristatin class of ADCs have been linked to high-grade neurological and ocular toxicities. Broadly, toxicities can be caused by both “on-target/ off-tumor” or “off-target/ off-tumor” toxicities; in addition, these toxicities may occur with or without AEs related to the payload itself (though ‘off- target, off- tumor’ AEs seem to dominate the toxicity profiles of most existing ADCs)^88,89^. Thus, expression pattern of the target antigen of inevitably influences the distribution of the payload and where it then accumulates. However meta-analyses have actually shown that MMAE is associated with anemia, neutropenia, and peripheral neuropathy independent of the target antigen; similarly, DM1 is associated with thrombocytopenia and hepatotoxicity, and MMAF and DM4 are associated with ocular toxicity, all irrespective of the target antigen^86,90,91^. Toxicities are not always predictable—for example, in spite of having the same payload, linker, and similar DARs, brentuximab vedotin and enfortumab vedotin have different toxicity profiles^88^. Trastuzumab deruxtecan and trastuzumab duocarmycin are both HER2-targeting ADCs that use different payloads, however, both cause pulmonary toxicities via an unknown mechanism; such toxicities have been observed, albeit to a lesser extent, with T- DM1^71,92,93^.

Destiny-lung01 reports ILD/pneumonitis at 26% with 6.4 kg/mg 3 weekly dosing^51^, however, a pulled analysis of nine T-DXd monotherapy studies evaluated ILD/pneumonitis risk in 1150 heavily pre-treated patients, and T-Dxd demonstrated a 15.4% rate of ILD with 2.2% grade 5 due to ILD in over 1,000 pts with breast and lung cancers^94^. In the most recent DESTINY-LUNG02 trial, which investigated 5.6 kg/mg dosing, the trial eligibility requires no current of history of ILD, no pneumonectomy, and no suspected ILD. Therefore, the use of T-Dxd needs to be cautioned for lung cancer patients with ILD or suspected ILD.

In lung cancer patients, deaths related to interstitial lung disease (ILD) have been reported with variable incidence in ADC clinical trials, and one can imagine this number may be higher in real-world settings. Appropriate training for physicians in the identification and management of this toxic and potentially deadly effect is urgently needed^95^. Generally, in symptomatic ILD the ADC should be discontinued; the re-introduction of the ADC can considered in asymptomatic cases following complete resolution and corticosteroids remain the cornerstone of ILD treatment. Steroid dosing depends on the severity of the event, and early diagnosis and treatment is key, as is the involvement of our pulmonary physician colleagues^95^. Early and accurate diagnosis of ILD is a significant challenge in the clinic, though recent developments in technology have led to Breath Analysis being used as a promising tool for ILD diagnosis. One study has demonstrated eNose technology, which uses breath analysis, could accurately identify ILD patients from a healthy control cohort, and in addition, could delineate between differing subgroups of ILD, suggesting a possible future biomarker in ILD which could identify ILD at an earlier stage of disease^96^. Due to the frequency of pulmonary toxicities such as ILD, using ADCs in patients with advanced metastatic lung cancer, caution is required, together with a high level of vigilance toward these and other rare, but potentially fatal, events. Thus, the toxicity profile of ADCs should be always considered when considering patients for treatment with ADCs.

## Future challenges and considerations

As ADCs are adopted into clinical practice, an understanding of resistance mechanisms will likely be crucial for future drug development, but due to the relative immaturity of the field of ADCs in lung cancer, clinical data on the resistance mechanisms is scant. However, pre-clinical data can provide potential insights to be considered. For example, in pre-clinical breast cancer models, following chronic exposure to TDM1, there was reduced cell surface HER2 expression with less TDM1 binding^97^. Proteomic analysis also identified upregulation of drug efflux pumps in these TDM1-resistant cell lines^97^, while transformed mechanisms of endocytosis with dysregulated ADC trafficking to lysosomes have also been recognized in TDM1-resistant cells^97,98^. In addition, pre-clinical NSCLC models have demonstrated loss of SLC46A3 expression as a mechanism of acquired resistance to DM1 (emtansine payload)^99^, and high expression of TUBB3 and FOXO3α in NSCLC has correlated with resistance to taxane-based ADC payloads^100^.

Another area of active investigation is patient selection. The success of TROP2 ADCs confirmed that ADCs are not just for oncogene-driven lung cancer, and that antigen expression is not necessarily a key feature required for the success of an ADC. The expression level of antigens on the surface of the cell is a continuous variable, which raises the question of whether patients should be pre-screened and excluded from treatment based on the presence of an antigen at a particular level. Optimal selection of patients for clinical trials evaluating ADCs is still uncertain, and some ongoing trials have adopted a prescreening phase to enroll only patients expressing the specific target, while others are limiting inclusion criteria to tumor types with a specific level of high target expression. ADCs, such as T-DXd, have induced responses in patients with reduced target expression, and there is clearly a critical need to develop validated assays and cutoffs to define antigen positivity, as well as predictive biomarkers of response.

Moving forward, rationale combination strategies will likely be important to augment ADC activity and overcome potential mechanisms of resistance. Trials of ADCs in combination with other anticancer therapies are already underway, from antiangiogenic agents, aiming to modify tumor vasculature and improve delivery to tumor tissues, to immunotherapy agents, which may have the potential to increase anti-tumor immunity induced by ADCs, by boosting cell-mediated tumor recognition and immune effector function the cytotoxic effects of ADC or by enhancing antibody-dependent cellular cytotoxicity^101^. Using these agents in combination with ADCs may increase the cell surface expression of the target antigen on the tumor cell and promote antibody–antigen engagement which may improve antigen turnover or degradation^101^.

To extend the therapeutic window of ADCs, site-specific ADCs, bispecific ADCs, or prodrug type ADCs are all being developed. Rapid developments in technology are driving these efforts: pClick technology, for example, is being developed to allow for site-specific conjugation^102^. pClick technology may allow for a new, more convenient, and more effective option to perform site-specific conjugation for the ADC development^102^. Bispecific antibody technology allow for intriguing ADC designs which may improve the internalization of the ADC and improve tumor specificity. Bispecific ADCs, which target different sites on the same tumor antigen, for example, could result in improved receptor aggregation and faster target internalization of the target^103,104^.

Dual-payload ADCs are another method being used to overcome potential mechanisms of resistance. These ADCs cleverly use two separate payloads, with different mechanisms of action and rationally, by using these two synergetic payloads in a controlled way, delivered into cancer cells could provide a more potent cytotoxic response^105^. One such example is an anti-HER2 ADC which contains MMAE and MMAF, which has shown promising efficacy in pre-clinical animal models^106^.

Payload alternatives to cytotoxic agents are also under development. Immunotoxins and Bcl-2 inhibitors are examples of payloads that, rather than being cytotoxic, induce apoptosis. ABBV-155, for example, targets B7-H3 and has clezutoclax as its payload, which induces apoptosis by inhibiting Bcl-XL. ABBV-155 is currently being investigated in a single agent and combination strategies using docetaxel or paclitaxel in a phase I trial, with expansion cohorts planned for small cell lung cancer and NSCLC (NCT03595059). LMB-100 is another experimental ADCs that incorporates pro-apoptotic payload, pseudomonas exotoxin A, which induces apoptosis by inhibiting elongation factor-2^107^. Immunomodulatory payloads are another intriguing concept: these non-cytotoxic payloads work by activating myeloid antigen-presenting cells and stimulating the immune system using a mechanism separate from checkpoint blockade^108^. SBT6050, for example, is an ADC targeting HER2 with a payload that is a toll-like receptor 8 (TLR8) agonist^31^. Novel technologies in the construction of ADCs, including alternatives to monoclonal antibodies (e.g., Nanobodies, protein scaffolds (designed ankyrin-repeat proteins (DARPins), and others), are also of interest.

## Conclusions

Following decades of research, considerable technological advances, and an improved understanding of the mechanism of action, ADCs are beginning to deliver on their initial promise^109–111^. In lung cancer, two ADCs have been granted FDA Breakthrough Therapy Designation and are currently under evaluation (patritumab deruxtecan, telisotuzumab vedotin) and one ADC has been granted accelerated approval (Fam-trastuzumab deruxtecan-nxki (T-DXd). With hundreds of ADCs in pre-clinical and clinical development across tumor types, the field shows no signs of slowing pace. In advanced lung cancer, ADCs have transformative potential for patients with limited treatment options. Ongoing clinical trials continue to assess novel ADCs, either as monotherapy or in combination strategies in lung cancer. Thus, the continued success of ADCs in the clinic may be inevitable, with the dawn of another paradigm shift in lung cancer on the horizon.

## Data Availability

Data referenced in this review can be accessed by following resources numbered in the Reference section.
